# COVID-19 vaccination of patients with allergies and type-2 inflammation with concurrent antibody therapy (biologicals) – A Position Paper of the German Society of Allergology and Clinical Immunology (DGAKI) and the German Society for Applied Allergology (AeDA) 

**DOI:** 10.5414/ALX02241E

**Published:** 2021-04-01

**Authors:** Oliver Pfaar, Ludger Klimek, Eckard Hamelmann, Jörg Kleine-Tebbe, Christian Taube, Martin Wagenmann, Thomas Werfel, Randolf Brehler, Natalija Novak, Norbert Mülleneisen, Sven Becker, Margitta Worm

**Affiliations:** 1 Department of Otorhinolaryngology, Head and Neck Surgery, Section of Rhinology and Allergy, University Hospital Marburg, Philipps-Universität Marburg,; 2 Center for Rhinology and Allergology, Wiesbaden,; 3 University Hospital for Pediatrics and Adolescent Medicine, Children’s Center Bethel, University of Bielefeld,; 4 Allergy Center Westend, Berlin,; 5 Clinic of Pneumology University Hospital Essen – Ruhrlandklinik, Essen,; 6 Clinic of Otolaryngology, University Hospital Düsseldorf, Düsseldorf,; 7 Department of Dermatology, Allergology and Venerology, Hannover Medical School, Hannover,; 8 Department of Dermatology, Wilhelm University of Münster, Münster,; 9 Department of Dermatology and Allergology, Bonn,; 10 Allergy and Asthma Center, Leverkusen,; 11 Department of Otorhinolaryngology, University Hospital, Tübingen, and; 12 Allergology and Immunology, Department of Dermatotology, Venereology and Allergology, Charité Universitätsmedizin Berlin, Germany

**Keywords:** allergy, antibodies, atopy, biologicals, COVID-19, vaccination

## Abstract

Background: After the beginning and during the worldwide pandemic caused by the severe acute respiratory syndrome coronavirus type 2 (SARS-CoV-2), patients with allergic and atopic diseases have felt and still feel insecure. Currently, four vaccines against SARS-CoV-2 have been approved by the Paul Ehrlich Institute in Germany, and vaccination campaigns have been started nationwide. In this respect, it is of utmost importance to give recommendations on possible immunological interactions and potential risks of immunomodulatory substances (monoclonal antibodies, biologicals) during concurrent vaccination with the approved vaccines. Materials and methods: This position paper provides specific recommendations on the use of immunomodulatory drugs in the context of concurrent SARS-CoV-2 vaccinations based on current literature. Results: The recommendations are covering the following conditions in which biologicals are indicated and approved: 1) chronic inflammatory skin diseases (atopic dermatitis, chronic spontaneous urticaria), 2) bronchial asthma, and 3) chronic rhinosinusitis with nasal polyps (CRSwNP). Patients with atopic dermatitis or chronic spontaneous urticaria are not at increased risk for allergic reactions after COVID-19 vaccination. Nevertheless, vaccination may result in transient eczema exacerbation due to general immune stimulation. Vaccination in patients receiving systemic therapy with biologicals can be performed. Patients with severe asthma and concomitant treatment with biologicals also do not have an increased risk of allergic reaction following COVID-19 vaccination which is recommended in these patients. Patients with CRSwNP are also not known to be at increased risk for allergic vaccine reactions, and continuation or initiation of a treatment with biologicals is also recommended with concurrent COVID-19 vaccination. In general, COVID-19 vaccination should be given within the interval between two applications of the respective biological, that is, with a time-lag of at least 1 week after the previous or at least 1 week before the next biological treatment planned. Conclusion: Biologicals for the treatment of atopic dermatitis, chronic spontaneous urticaria, bronchial asthma, and CRSwNP should be continued during the current COVID-19 vaccination campaigns. However, the intervals of biological treatment may need to be slightly adjusted (DGAKI/AeDA recommendations as of March 22, 2021).

## Introduction 

[Table Abbreviations]During March 2020, the World Health Organization (WHO) declared a pandemic for severe acute respiratory syndrome coronavirus type 2 (SARS-CoV-2)-transmitted corona virus infectious disease 19 (COVID-19) [1]. Since then, numerous position papers and practical guidance for optimal care of patients have been published by international and national allergy societies [2, 3, 4, 5, 6]. In addition, recommendations for the use of immunomodulatory antibodies (biologicals) and allergen immunotherapy (AIT) products were formulated early during the pandemic and adapted to the German healthcare situation [7, 8, 9]. 

At present, two mRNA-based vaccines (*Comirnaty* from BioNTech [10] and *COVID-19 Vaccine* from Moderna [11]) and two vector-based vaccines (*Vaxzevria* from AstraZeneca [12] and *COVID-19 Vaccine* from Johnson & Johnson [13]) have been approved by the European Medicines Agency (EMA) in Europe. Vaccination campaigns were initiated at the end of December 2020 in Germany. An interdisciplinary expert group “Management of Anaphylaxis” formed by the German Society of Allergology and Clinical Immunology (DGAKI), the German Society for Applied Allergology (AeDA), and the Society for Pediatric Allergology and Environmental Medicine (GPA) have published recommendations for the risk assessment of allergic reactions during COVID-19 vaccinations [14, 15]. Moreover, practical guidance has been provided for the management of patients at risk of anaphylaxis [16]. 

According to the Summary of Product Characteristics (SmPC) of the four authorized COVID-19 vaccines in Europe, immunosuppressive or immunomodulatory therapies including biologicals are not contraindicated, but it is noted in the SMPC’s leaflet that *“efficacy may be lower in immunosuppressed patients”* [10, 11, 12, 13]. On this basis, learned societies have issued preliminary recommendations for the use of biologicals during concurrent COVID-19 vaccinations. In a first *ad hoc* statement, the DGAKI advocates the concurrent use of monoclonal antibodies (benralizumab, dupilumab, mepolizumab, omalizumab, and reslizumab) and COVID-19 vaccines [17]. In line with a statement of the German Standing Committee on Vaccination (Ständige Impfkommission (STIKO)) on inactivated vaccines and concurrent immunomodulatory therapy [18], the German allergological societies published a joint declaration to schedule the COVID-19 vaccination in the middle of a therapy interval of biological treatment [17, 19, 20]. 

**Figure 1.**
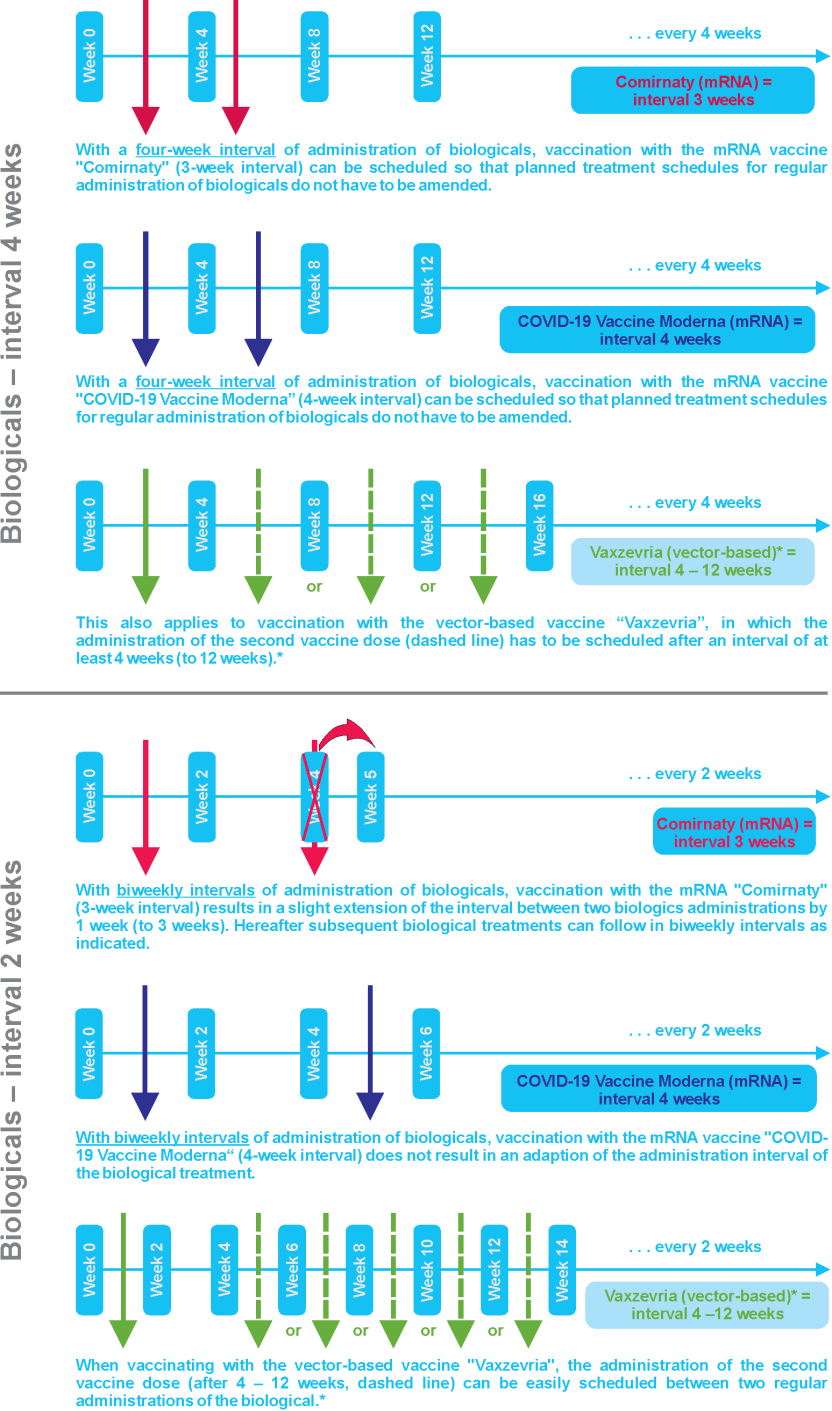
Recommendations for time intervals between COVID-19 vaccines and biologicals. *The vector-based vaccine “COVID-19 Vaccine Janssen” is administered as a single-dose only.

The aim of this position paper is to outline practical implications for the concurrent use of biologicals in different indications such as atopic dermatitis, chronic spontaneous urticaria, bronchial asthma, and chronic rhinosinusitis with nasal polyps (CRSwNP) with COVID-19 vaccination and to provide recommendations for best practice management (Table 1, Figure 1).

## Chronic inflammatory skin diseases 

Chronic inflammatory skin diseases such as atopic dermatitis and chronic spontaneous urticaria are commonly treated with systemic immunomodulatory therapy in severe courses, which arises concern about potential risks of COVID-19 infection and vaccination under this systemic therapy [21]. 

Patients with atopic dermatitis are not at increased risk for allergic reactions to COVID-19 vaccination. Nevertheless, vaccination may result in transient eczema exacerbation due to general immune stimulation. 

Topic anti-inflammatory local therapy with both steroids and calcineurin inhibitors does not influence the efficacy of vaccination. Besides, patients receiving systemic therapy with ciclosporin, methotrexate, azathioprine, or baricitinib can be vaccinated at any time. However, the effect of vaccination may be reduced by the systemic immunosuppression [22, 23]. Therefore, the European Task Force on Atopic Dermatitis of the European Academy of Dermatology and Venereology (EADV) recommends either a temporary interruption of treatment or a reduced dosage of the aforementioned substances (details in [24]). 

Patients receiving systemic therapy with dupilumab can also be vaccinated at any time. Data from a previous study of patients treated with dupilumab receiving vaccination with tetanus toxoid demonstrated that the efficacy of the vaccination was not adversely affected by dupilumab treatment [25]. Currently, for practical reasons, it is recommended that vaccination be administered between dupilumab injections. If specific time intervals need to be observed regarding vaccination, dupilumab injections should be adjusted accordingly [24]. In general, COVID-19 vaccination should be applied in the treatment interval between two injections of the biological, that is, with a time-lag of at least 1 week after the previous or at least 1 week before the next biological treatment planned. This recommendation also applies to the biologicals listed below. 

Patients with chronic spontaneous urticaria also have no increased risk of allergic reactions to COVID-19 vaccinations. Nevertheless, vaccination may result in transient exacerbation due to general immune stimulation. Systemic antihistamines do not impact the effect of the vaccination. Patients treated with systemic steroids and/or ciclosporin can be vaccinated at any time. However, the effect of vaccination may be reduced by this systemic immunosuppression as well, so immunological response to COVID-19 vaccination should be verified by serum antibody levels, if applicable. Patients treated with omalizumab can be vaccinated at any time. As with other biologicals, it is recommended that vaccination be administered between omalizumab injections (see above) and slight amendments of the intervals for biological treatment may be followed as stated above. 

## Bronchial asthma 

For the treatment of severe uncontrolled bronchial asthma, biologicals are approved as an add-on treatment option to standard anti-inflammatory treatment with inhaled corticosteroids + long-acting β-agonists (LABA) and long-acting muscarinic antagonists (LAMA). They are targeting key mechanisms of the inflammatory cascade in bronchial asthma with high efficacy and a good safety profile in this indication. In this respect, it is of utmost importance to further focus on possible immunological interactions of biologicals during concurrent vaccination with the approved vaccines. 

The asthmatic inflammatory response is based on the T2 immune response, in which immunoglobulin E (IgE) and the cytokines interleukin (IL-)4, IL-5, and IL-13 in particular are key elements targeted by biologicals. It is important to note that neither IgE nor the aforementioned cytokines play a role in the antiviral immune response. Study data on vaccine responses to other antiviral vaccines and concurrent treatment with biologicals are also available with no hint for a blunting of the immune response by these vaccinations [25, 26]. Moreover, previous studies have demonstrated that anti-IgE treatment indeed increases the type-1 interferon production, thereby enhancing antiviral response [27, 28]. Preliminary data in asthmatic patients with a COVID-19 infection indicate no difference in antibody production against SARS-CoV2 in patients with concurrent biological treatment in comparison to patients without this therapy [29]. 

As a general rule, a risk/benefit assessment must be followed for all vaccines and concurrent treatment with biologicals. In patients with severe asthma and concurrent biological therapy, vaccination against SARS-CoV-2 is recommended, and the intervals for biological treatment may be slightly amended as stated above. 

## Chronic rhinosinusitis with nasal polyps 

To date, there is no evidence that patients with chronic rhinosinusitis (with or without nasal polyps) may have an increased likelihood of allergic reactions following any of the various COVID-19 vaccines. This holds also true for susceptibility to SARS-CoV-2 infection or severity of COVID-19 disease [30]. 

In the majority of patients with CRSwNP, a type-2 directed inflammation is the underlying pathomechanism involved with multiple inflammatory pathways [31, 32]. As those are also included in the inflammatory pathways of bronchial asthma, most of the antibodies approved in this indication have also been shown to be effective in CRSwNP [33]. 

As an add-on treatment for severe, uncontrolled forms of CRSwNP, two biologicals have been recently approved in Germany (dupilumab and omalizumab). Based on preliminary trial data, two additional antibodies may gain market authorization in due time (mepolizumab and benralizumab). The application and dosage of these four preparations are identical to those for asthma therapy. Therefore, the same considerations regarding their compatibility with COVID-19 vaccinations specified above for bronchial asthma also apply in principle to biological treatment of CRSwNP. 

In line with the recommendations outlined above, continuation or initiation of a biological treatment for CRSwNP is recommended with a concurrent planning of a COVID-19 vaccination. For the biologicals indicated in CRSwNP, general recommendations regarding the interval between vaccinations and biological therapies as outlined above should be followed [17, 19, 20]. 

## Conclusion 

Currently, there is no evidence of an increased allergic risk from COVID-19 vaccinations in patients with chronic inflammatory skin diseases (atopic eczema, chronic spontaneous urticaria), bronchial asthma, and CRSwNP. Approved immunomodulatory biologicals with proven high efficacy and safety are indicated in uncontrolled courses of these diseases. 

These therapies should not be temporarily interrupted due to a planned COVID-19 vaccination as they are not contraindicated for these vaccines. However, a COVID-19 vaccination should be given within the interval between two applications of the respective biological, that is, with a time-lag of at least 1 week after the previous or at least 1 week before the next biological treatment planned. 

## Funding 

None. 

## Conflict of interest 

O. Pfaar reports grants and/or honoraria from ALK-Abelló, Allergopharma, Stallergenes Greer, HAL Allergy Holding B. V./HAL Allergie GmbH, Bencard Allergie GmbH/Allergy Therapeutics, Inmunotek S.L., Lofarma, Biomay, Circassia, ASIT Biotech Tools S. A., Laboratorios LETI/LETI Pharma, MEDA Pharma/MYLAN, Anergis S. A., Mobile Chamber Experts (a GA2LEN Partner), Indoor Biotechnologies, GlaxoSmithKline, Astellas Pharma Global, EUFOREA, ROXALL Medizin, Novartis, Sanofi Aventis, Sanofi Genzyme, Med Update Europe GmbH, streamedup! GmbH, Pohl-Boskamp GmbH, John Wiley and Sons AS, Paul Martini Foundation (PMS), during the last 36 months and all outside the present work. 

L. Klimek reports grants and/or honoraria from Allergopharma, Bioprojet, Viatris, HAL Allergie, ALK Abelló, Aimmune, LETI Pharma, Stallergenes, Quintiles, Sanofi, ASIT Biotech, Lofarma, Thermofisher, Roxall, Allergy Therapeutics, AstraZeneca, GSK, Inmunotek, outside the submitted work; and membership in the following organizations: AeDA, DGHNO, German Academy of Allergology and Clinical Immunology, German Allergy League; ENT-BV, GPA, EAACI. 

R. Brehler reports honoraria from ALK Abelló, Allergopharma, Allmiral, AstraZeneca, Bencard, Gesellschaft zur Förderung der Dermatologischen Forschung und Fortbildung e.V., GSK, HAL Allergie, LETI Pharma, MedUpdate, Merck, Novartis, Sanofi, Stallergenes, outside the submitted work; and membership in the following organizations: AeDA, DGAKI, EAACI, ABD. 

M. Wagenmann reports honoraria from ALK-Abello, Allergopharma, AstraZeneca, Bencard, Genzyme, GlaxoSmithKline, HAL Allergy, LETI, Meda Pharma, Novartis, Sanofi Aventis, Stallergenes, all outside the present work. 

N. Novak reports honoraria from Alk Abello, Stallergens Geer, Hal Allergy, Leti Pharma, Sanofi Genzyme, Abbvie, Leo Pharma, Novartis, streamed up und Blueprint, all outside the present work. 

M. Worm reports conflicts of interest by Regeneron Pharmaceuticals, DBV Technologies S.A, Stallergenes GmbH, HAL Allergy GmbH, Bencard Allergie GmbH, Allergopharma GmbH & Co. KG, ALK-Abelló Arzneimittel GmbH, Mylan Germany GmbH, Leo Pharma GmbH, Sanofi-Aventis Deutschland GmbH, Aimmune Therapeutics UK Limited, Actelion Pharmaceuticals Deutschland GmbH, Novartis AG, Biotest AG, AbbVie Deutschland GmbH & Co. KG, Lilly Deutschland GmbH – all outside the present work. 

The other authors declare no conflicts of interest. 

**Abbreviations Abbreviations:** Abbreviations.

AeDA	German Society for Applied Allergology (Aerzteverband deutscher Allergologen)
COVID-19	Corona virus infectious disease 19
CRSwNP	Chronic rhinosinusitis with nasal polyps
DGAKI	German Society of Allergology and Clinical Immunology (Deutsche Gesellschaft für Allergologie und Klinische Immunologie)
GPA	Society for Pediatric Allergology and Environmental Medicine (Gesellschaft für Pädiatrische Allergologie und Umweltmedizin)
EADV	European Academy of Dermatology and Venereology
EMA	European Medicines Agency
IgE	Immunoglobulin
IL	Interleukin
LABA	Long-acting β-agonists
LABA	Long-acting muscarinic antagonists
SARS-CoV2	Severe acute respiratory syndrome coronavirus type 2
SmpC	Summary of Product Characteristics
STIKO	(German) Standing Committee on Vaccination (Ständige Impfkommission)
WHO	World Health Organization

**Table 1.** DGAKI/AeDA recommendations (as of March 22, 2021).

Diseases	Recommendations on COVID-19 vaccines	Recommendations on COVID-19 vaccines and biologicals
Atopic dermatitis	No increased risk of allergic reactions to COVID-19 vaccination. Vaccination possible at any time. Short-term eczema aggravation possible due to vaccination.	Vaccination can be applied at any time under dupilumab. Vaccination is recommended between two dupilumab injections with 1 week interval between vaccination and this biological treatment.
Chronic spontaneous urticaria	No increased risk of allergic reactions to COVID-19 vaccination. Vaccination possible at any time. Short-term eczema aggravation possible due to vaccination.	Vaccination can be applied at any time under omalizumab. Intervals between vaccinations and biological therapies as outlined above should be followed.
Bronchial asthma	No increased risk of allergic reactions to COVID-19 vaccination. Vaccination possible at any time.	Vaccination is recommended in patients with severe asthma and concurrent biological treatment. Intervals between vaccinations and biological therapies as outlined above should be followed.
Chronic rhinosinusitis with polyps (CRSwNP)	No increased risk of allergic reactions to COVID-19 vaccination. Vaccination possible at any time.	Continuation or initiation of biological therapy in CRSwNP is recommended (if indicated) with concurrent vaccination. Intervals between vaccinations and biological therapies as outlined above should be followed.
